# Therapeutic vaccination of koalas harbouring endogenous koala retrovirus (KoRV) improves antibody responses and reduces circulating viral load

**DOI:** 10.1038/s41541-020-0210-9

**Published:** 2020-07-16

**Authors:** Olusola Olagoke, Bonnie L. Quigley, Farhid Hemmatzadeh, Galit Tzipori, Peter Timms

**Affiliations:** 1 Genecology Research Center, Faculty of Science, Health, Education and Engineering, University of the Sunshine Coast, 90 Sippy Downs Drive, Sippy Downs, QLD 4556 Australia; 2 School of Animal and Veterinary Sciences, The University of Adelaide, Roseworthy, SA 5371 Australia; 3 Lone Pine Koala Sanctuary, Fig Tree Pocket, Queensland, Australia

**Keywords:** Vaccines, Retrovirus, Infectious diseases

## Abstract

The long-term survival of the koala is under serious threat from multiple factors, including infectious disease agents such as *Chlamydia* and koala retrovirus (KoRV). KoRV is present in both exogenous and endogenous forms, depending on the geographical location of the population. In the northern half of Australia, it is present as an endogenous infection in all koalas, making a case for an urgent need to develop a therapeutic vaccine that might prevent KoRV-associated pathologies in these koalas. To this end, we determined the therapeutic effects of vaccinating koalas harbouring endogenous KoRV with a recombinant KoRV Env protein combined with a Tri-adjuvant. We found that vaccination led to a significant increase in circulating anti-KoRV IgG levels, as well as increase in neutralising antibodies. Our study also showed that post-vaccination antibodies were able to recognize epitopes on the Env protein that were unrecognised pre-vaccination, as well as resulting in an increase in the recognition of the previously recognised epitopes. The vaccine also induced antibodies that were cross-reactive against multiple KoRV-subtypes. Finally, we found a complete clearance of KoRV-A in plasma from koalas that had detectable levels of KoRV-A pre-vaccination. Similarly, there was a significant reduction in the expression of KoRV-B viral RNA levels post-vaccination. Collectively, this study showed that koalas harbouring endogenous KoRV can benefit from prophylactic vaccination against KoRV using a recombinant KoRV-A Env protein and that the mechanism of this protection might be through the boosting of natural anti-KoRV antibodies and expanding the breadth of the recognised epitopes.

## Introduction

Koala retrovirus (KoRV) has been linked to lymphoma, leukaemia, immunosuppression and increased susceptibility to chlamydial disease in both wild and captive koala populations and is suspected to contribute a significant threat to the long-term survival of this iconic species^1–3^. Though KoRV was only discovered 20 years ago^4^, current testing shows that all northern koalas (Queensland and New South Wales) are infected with KoRV^5^. Nine KoRV subtypes (KoRV-A to KoRV-I) have been identified based on variations within the receptor binding domain (RBD) of the envelope (Env) protein, with KoRV-A and KoRV-B being the most widely studied^6,7^. While KoRV-A uses a phosphate transporter (PiT1), KoRV-B uses the thiamine transporter 1 (ThTR1) for cell attachment and entry^1,8^. Northern koalas are believed to harbour endogenous KoRV-A as determined by consistent genome integration patterns in family groups as well as high proviral copies^9^. In addition, KoRV-A also shows patterns consistent with that of an infectious exogenous retrovirus as shown by the presence of replication competent KoRV-A in northern koalas^10^. Unlike KoRV-A, other KoRV subtypes are thought to exist either as exogenous or defective viruses^1,9^. The existence of KoRV as both an endogenous and an exogenous virus in northern koalas suggests it can be transmitted both vertically and horizontally.

A strong link has been suggested to exist between KoRV infection and koala pathologies in both captive and wild koalas. For instance, KoRV has been linked with increased chlamydial disease in northern koalas^3,11^. While the definitive level at which KoRV becomes harmful to koala health is currently unknown, current studies suggest that a significant increase in KoRV viral load is positively associated with koala pathologies such as neoplasia^2^. As such, there is an urgent need for treatment options that may lead to reduction in KoRV viral load. While antiretroviral therapies are possible options, these are not practical in wild koala populations. This leaves vaccination as the only realistic KoRV management option.

Efficacious vaccines can be used to induce protective immunity where the natural immune response is inadequate. This is especially true where vaccines are designed to present viral epitopes not normally recognised in natural infection. Following the successful vaccination of rats and goats with recombinant KoRV Env protein, it was suggested that koalas may benefit from vaccination against KoRV^12^. This hypothesis was proven recently when southern koalas (South Australia) were shown to produce neutralising antibodies following vaccination with a recombinant KoRV Env protein^13^. The vaccine was also shown to reduce viral load in the KoRV positive group.

In the current study, we investigated the possibility of vaccinating koalas harbouring endogenous KoRV with a recombinant KoRV-A Env protein. We determined if the vaccine could induce an immune response against multiple KoRV subtypes. We showed that vaccination induced strong anti-KoRV antibody titres that were capable of neutralising KoRV in vitro. We also demonstrated a reduction in the expression of KoRV in plasma following vaccination. This study showed that the antibody produced post-vaccination was cross-reactive against KoRV-B.

## Results

### Anti-KoRV antibody titre is significantly increased following vaccination

**Fig. 1:**
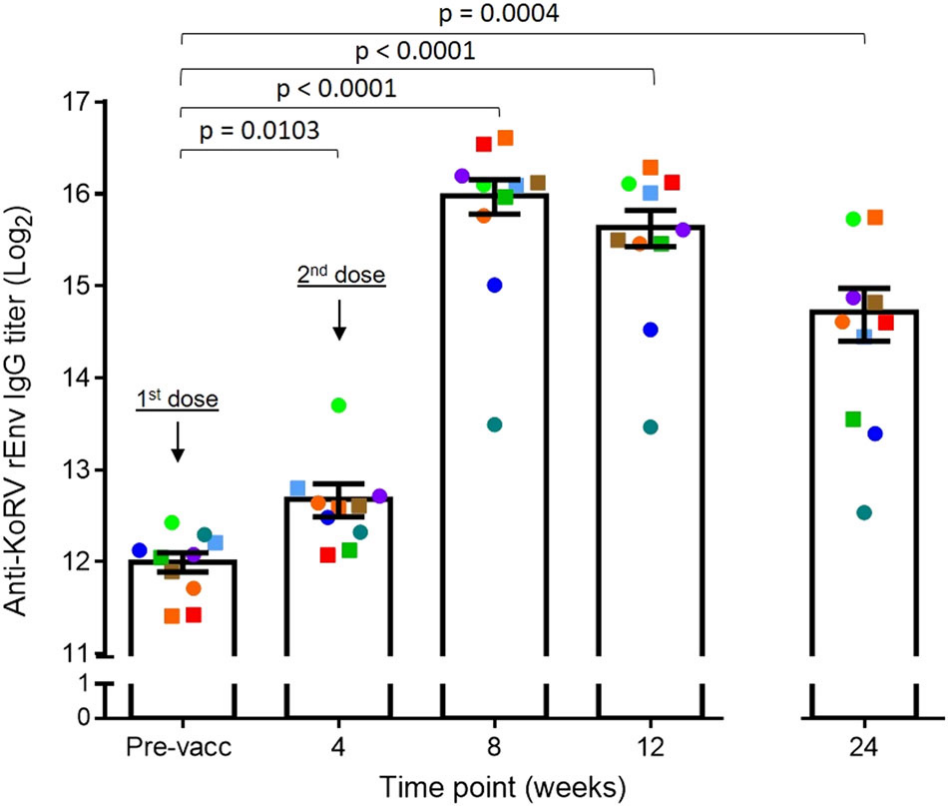
Anti-KoRV IgG levels expressed as end point titres (Log2) in vaccinated koalas (*n* = 10) harbouring endogenous KoRV. Data are as the mean ± SEM.

We recently showed that naturally infected koalas harbouring endogenous KoRV make anti-KoRV antibodies^14^. In the current study, we evaluated whether a recombinant KoRV Env protein vaccine was able to induce anti-KoRV antibodies in koalas harbouring endogenous KoRV. Ten captive northern koalas (endogenously infected with KoRV-A) were vaccinated subcutaneously with two doses of the vaccine. Prior to vaccination, we detected anti-KoRV IgG titres that were similar to levels previously reported in wild koalas harbouring endogenous KoRV^14^. Following vaccination, all animals had significant increases in total anti-KoRV IgG levels, ranging from a 2–37 fold increase by week 8 (Fig. 1). The antibody titres peaked around week 8 to 12, with a slight decline in all animals by 24 weeks post-vaccination. At 24 weeks post-vaccination, anti-KoRV antibody levels were still 3–20 fold above pre-vaccination levels.

### Reduction in KoRV viral RNA expression levels following vaccination

**Fig. 2:**
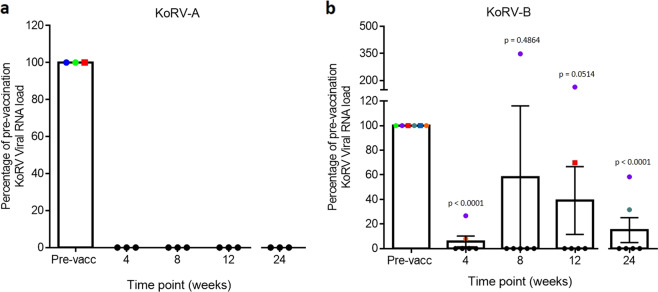
Change in KoRV viral RNA load following vaccination in koalas as measured by qPCR. Expression at each time point were compared against pre-vaccination levels and presented as mean ± SEM. The level of significance was measured using Student’s *T* test (≤0.05). The coloured shapes represent individual koalas with detectable KoRV expression. These are subsequently represented with black circles when KoRV expression becomes undetectable.

To determine the therapeutic potential of vaccinating koalas harbouring endogenous KoRV-A with a recombinant KoRV-A Env protein-based vaccine, we measured circulating viral load in plasma at pre-vaccination and weeks 4, 8, 12 and 24 post-vaccination. Three out of 10 vaccinated koalas were found to have detectable levels of circulating KoRV-A *env* gene expression in plasma pre-vaccination (Fig. 2a). Following vaccination, there was a complete clearance of KoRV-A in plasma from those three koalas (Fig. 2a).

The vaccine used in this study was based on the KoRV-A Env protein sequence (amino acids 98 to 657 of the KoRV-A envelope protein; AHY24807.1). KoRV-B, which has been implicated in koala pathologies such as lymphoma and chlamydial disease, shares significant sequence similarity with KoRV-A. Amino acid differences between KoRV-A and KoRV-B envelope proteins are limited to ~40 amino acids, with 36 of these differences occurring within the RBD (Supplementary Fig. 1). We therefore determined if the KoRV-A based vaccine could lead to reduction in KoRV-B expression levels in plasma. KoRV-B *env* gene expression was detectable in six out of 10 koalas prior to vaccination (Fig. 2b). In all six KoRV-B expressing koalas, the expression of KoRV-B viral RNA was significantly reduced by four weeks post-vaccination. For five of these koalas, KoRV-B expressed RNA levels remained undetectable at 8 weeks. At both 12 and 24 weeks post-vaccination, KoRV-B expression remained undetectable in four out of six koalas. One animal was an outlier in that it had a significant increase in its KoRV-B expression level at week 8 which was then followed by a gradual reduction at 12 and 24 weeks post-vaccination.

We also determined changes in the expression of two additional KoRV subtypes (KoRV-D and F; GenBank accession numbers AB828004.1 and KX587994.1 respectively) with no currently known associations with adverse health outcomes. We observed a general reduction in viral load in most koalas post-vaccination (Supplementary Fig. 2).

### Vaccination expands IgG recognition of KoRV-A Env protein in koalas harbouring endogenous KoRV-A

**Fig. 3:**
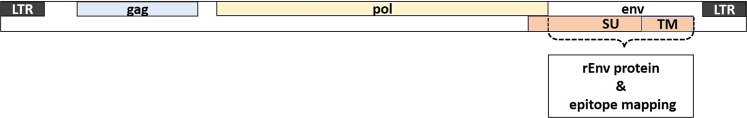
Schematics showing KoRV genome and the segments relevant to this study. SU surface protein subunit, TM transmembrane subunit.

To identify which amino acid sequences on the KoRV-A Env protein were antigenic and linked to the strongest IgG response in koalas harbouring endogenous KoRV, we designed 138 15mer overlapping peptides with 4 amino acids offsets. The overlapping peptides span the entire amino acids sequence of the KoRV-A Env protein used for the production of the recombinant vaccine antigen. The sequences used allowed for mapping of the entire transmembrane subunit and most of the surface protein unit (Fig. 3). Plasma samples were tested against each peptide at four timepoints; pre-vaccination (week 0), 8, 12 and 24 weeks post-vaccination.

**Fig. 4:**
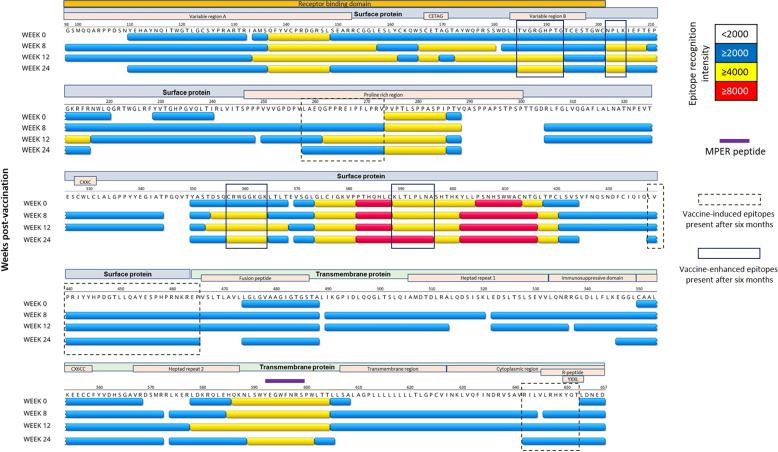
KoRV Env protein B-cell epitope mapping of koalas (*n* = 5) harbouring endogenous KoRV-A following vaccination. Data are shown on the amino acid sequence of KoRV-A envelope protein with amino acid scoring method. Coloured amino acids are regions recognised by koala IgG: blue, yellow and red represent an average of combined fluorescent intensity of equal to or greater than 2000, 4000 and 8000 respectively. Area colour code: gold: receptor binding domain; grey: surface protein; light green: transmembrane protein; orange: motifs and regions. Motifs: CETAG, CXXC, CX6CC and YXXL. MPER: membrane proximal external region.

At pre-vaccination, we found that antibodies recognised regions across 93 out of the 106 amino acids present within the RBD (Fig. 4). Within the RBD, the sequence QFYVCPRDGRSL (AA 137–148) was recognised with high intensity (Fig. 4, yellow regions). We also observed two additional highly antigenic regions within the SU subunit: PVPTLSPPASPI (AA 274–285), which falls within the proline rich region, and GLCIGKVPPTHQHLCKLTLPLNASHTHKYLLPSNHSWWACNTGL (AA 374–417) (Fig. 4, yellow regions). The underlined sequences were recognised with even higher intensity (Fig. 4, red regions). A final highly antigenic region was identified within the membrane proximal external region of the TM subunit QKNLSWYEGWFNRSPWLTL (AA 586–604) (Fig. 4, yellow regions).

Several regions not recognised in koalas prior to vaccination were recognised post-vaccination. At 8 and 12 weeks post-vaccination, 534 and 528 AA of the total 560 AA present in the vaccine antigen were recognised, respectively, compared to only 281 AA that were recognised pre-vaccination (Fig. 4, amount of sequence covered by blue, yellow and red bars at each time point). Three unique regions not recognised pre-vaccination were recognised by koala antibodies at all time points post-vaccination (Fig. 4, dashed boxes). These include the two sequences within the surface protein subunit; LAEQGPPREIPFLPRV, LVPRIYYHPDGTLLQAYESPHPRNKREP, and one sequence within the TM subunit; RILVLRHYQT (Fig. 4, dashed boxes). In addition, some regions that were recognised in natural infections were boosted following vaccination (Fig. 4, solid boxes). Four regions had a sustained increase in recognition out to 24 weeks post-vaccination. These included the sequence TVGRGHPTG, present within the Variable region B of the RBD; NPLK; CRWGGKGK and KLTLPLNA. All these sequences were present within the surface protein subunit of the Env protein (Fig. 4, solid boxes).

### Vaccination enhances IgG response to conserved and variable domains of the RBD of KoRV-B

**Fig. 5:**
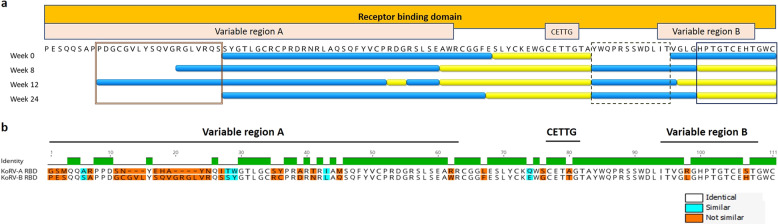
B-Cell Epitope mapping. **a** Epitope mapping of the receptor binding domain of KoRV-B in koalas (*n* = 5) harbouring endogenous KoRV-A following vaccination. Data are shown with amino acid scoring method. Coloured amino acids are sequences recognised by koala IgG: blue, yellow and red represent an average of combined fluorescent intensity of equal to or greater than 2000, 4000 and 8000 respectively. **b** Comparison of the receptor binding domain amino acid sequences of Env protein of KoRV-A and KoRV-B used in this study. Differences in amino acid sequences are highlighted.

To better understand the observed protection against the more pathogenic KoRV-B subtype, we determined if the KoRV-A Env protein-based vaccination induced antibodies that were cross-reactive against the RBD of KoRV-B Env protein. Plasma samples from 0, 8, 12 and 24 weeks post-vaccination were tested against 30, 15mer overlapping peptides with 4 amino acids offsets spanning KoRV-B RBD. The RBD was chosen for this assay because it contains most of the amino acid variations present between the KoRV-A and KoRV-B Env protein sequences. Prior to vaccination, we observed that koalas made antibodies that recognised both conserved and variable domains within the KoRV-B Env protein (Fig. 5a, blue and yellow bars at week 0). Following vaccination, the antibodies produced recognised a conserved region on the KoRV-B RBD which was previously unrecognised pre-vaccination (Fig. 5a, dashed box). We also observed that the antibodies produced following vaccination were cross-reactive against regions with minor changes on the KoRV-B RBD, such as the Variable region B (Fig. 5a, solid box). Finally, post-vaccination antibodies were cross-reactive against the region with major sequence differences, also known as the hypervariable region, on the KoRV-B RBD, only at weeks 8 and 12 but not 24 weeks (Fig. 5a, compound box at beginning of sequence and Fig. 5b).

### Vaccination increases KoRV-neutralising antibody titres

**Fig. 6:**
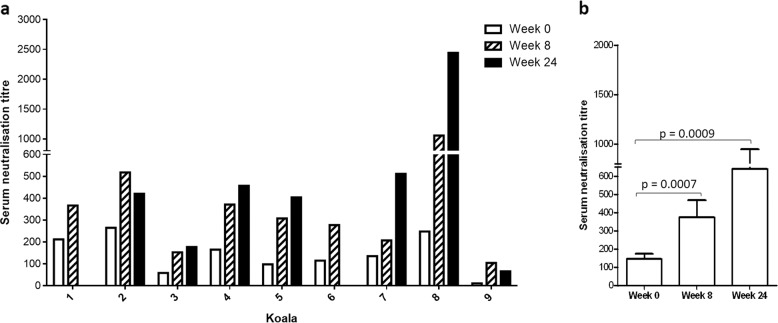
Neutralising antibodies. **a** KoRV-neutralising antibody activity of individual koala serum following vaccination measured at weeks 0, 8 and 24. Serum neutralisation titre was defined as the reciprocal of the serum dilution that resulted in 50% reduction in KoRV-A proviral integration when compared to the no serum control. **b** The average serum neutralisation titres at weeks 0, 8 and 24 in vaccinated koalas were compared and presented as mean ± SEM. The level of significance was measured using Student’s *T* test (*p* ≤ 0.05).

We examined if vaccinating koalas that harbour endogenous KoRV with a recombinant KoRV-A Env protein was capable of inducing KoRV-neutralising antibody. To do this, we infected human embryonic kidney (HEK) 293T cells with KoRV in the presence or absence of serially titrated koala sera (pre- and post-vaccination). Genomic DNA was subsequently extracted from the infected HEK293T cells and analysed for KoRV-A proviral integration levels. Serum neutralisation titre was defined as the reciprocal of the serum dilution that resulted in 50% reduction in KoRV-A proviral integration, when compared to a no serum control. Prior to vaccination, we observed that koala sera in general had some basal level of “neutralising” effect when tested in this in vitro assay (background titre ranging from 10 to 250). Post-vaccination, we observed significant increases in KoRV neutralisation levels in vitro. Vaccinated koalas produced increased in vitro neutralisation levels at 8 and or 24 weeks post-vaccination, compared to the zero time point, with these serum titres ranging from 100 to 1000, and 65 to 2500, respectively (Fig. 6a). Using the student’s *T* test (*p* ≤ 0.05), we conducted a summary statistic to compare the average neutralisation titre of sera from eight and 24 weeks post-vaccination to average pre-vaccination titre. Analysis showed that both eight and 24 weeks had significantly higher serum neutralisation activity when compared to pre-vaccination levels (Fig. 6b). The level of significance at weeks eight and 24 were measured as *t*(8) = 5.286, *p* = 0.0007, and *t*(6) = 4.049, *p* = 0.0009 respectively.

### MHC class I and II allele diversity in trial koalas

Koalas are outbred animals and we would expect a significant level of genetic diversity between the animals in this trial. We used MHC profiling to examine the immunogenetic variation in our vaccinated koalas and to determine if any within group differences in immune response parameters might be explained by host genetic differences. We targeted the previously described MHC class I (UA and UC) and MHC class II (DAb, DBb, DCb and DMb) gene alleles^15,16^. There were no major differences between the MHC profiles of the one weak vaccine responder (Clifton, denoted by a dark green circle in Fig. 1) and the remaining nine koalas in this study (Table 1). Between all the vaccinated koalas, we detected seven UA alleles and five UC MHC class I gene alleles (Table 1). For the MHC class II genes, we detected five different alleles for each of the DAb and DBb genes and four different alleles for each of the DCb and DMb genes amongst the vaccinated koalas (Table 1). While Clifton shared most of its MHC genotypes with other koalas, it had a unique combination of the MHC class II gene DBb (DBb*01,02).

**Table 1:** MHC class I and class II gene alleles showing genetic diversity of trial koalas.

Koala ID	MHC class II gene alleles				MHC class I gene alleles		Koala age (year)
	DAb	DBb	DCb	DMb	UA	UC	
Sargent	10, 15, 19, 37	1, 4	1, 3	2, 3	1:1, 13:1	5:2	6.2
Jester	15, 19, 37	2, 5	2, 3	2, 4	1:1, 8:1, 9:1	1:5, 5:1	2.3
Feenie	15, 19, 23, 37	2, 5	1	1	8:1, 11:1, 12:1	1:3	2.2
Fraggle	10, 15, 19, 37	1, 2, 5	1	1, 2	1:1, 8:1, 11:1	1:1, 1:3	3.5
Grover	10, 15, 19, 37	2, 4	1, 3	1, 2	8:1, 9:1, 13:1	1:1	3.6
Milton	10, 19, 37	1, 3	1	1, 3	8:1, 10:1, 13:1	1:1, 5:2	2
**Clifton**	**10, 15, 19, 37**	**1, 2**	**1**	**2, 3**	**8:1, 9:1, 13:1**	**1:1, 5:2**	**3.4**
Daiquari	15, 19, 23, 37	3	1, 3	1, 2	8:1, 9:1, 10:1	1:1	3.3
Davis	10, 15, 19, 37	2, 3, 4, 5	1, 2	1, 3	1:1, 8:1, 9:1	1:1, 1:3	3.4
Aster	15, 19, 23, 37	2, 4	1, 2	3, 4	8:1, 10:1, 11:1	1:1	6

The notable poor vaccine responder (based on antibody levels from Fig. 1) is shown in bold.

## Discussion

KoRV, along with *Chlamydia*, poses a significant threat to the continued survival of the koala. Since its discovery in 20004 KoRV has been linked with neoplasia, especially in captive koala populations, as well as increased chlamydial disease in wild koalas^2,3^. While there are established treatment regimens consisting of various antibiotics for chlamydial disease in affected koalas, there is currently no treatment available for KoRV. Any treatment developed must be of practical use in wild koalas which constitute most Australia’s koalas, thus making vaccination a very practical option. In addition to the presence of exogenous KoRV infection, all northern koalas (Queensland and New South Wales) harbour endogenous KoRV in their genome. We recently showed that vaccination with a recombinant KoRV-A Env protein led to the induction of neutralising antibodies and reduction in viral load in KoRV negative koalas and those believed to be exogenously infected with KoRV^13^. In the present study, we examined the effect of the recombinant KoRV-A Env protein-based vaccine in koalas harbouring endogenous KoRV. Our results show that vaccination had a therapeutic effect on koalas by inducing the production of neutralising antibodies and reducing KoRV viral load.

We previously showed that koalas harbouring endogenous KoRV are not tolerant to the KoRV but rather make antibodies against distinct regions on the KoRV Env protein^14^. This highlighted the possibility of developing a therapeutic vaccine to boost natural immunity. In the present study, we confirmed this hypothesis by showing that vaccinating koalas harbouring endogenous KoRV led to a significant increase in anti-KoRV IgG levels, for up to six months post-vaccination. Our findings greatly expand a pilot study that showed a single vaccinated koala with endogenous KoRV was found to produce anti-KoRV IgG against the KoRV transmembrane protein^17^. Due to assay limitations, that study could not determine if vaccination boosted pre-vaccination antibody titres. In our study, however, we show that vaccinating koalas harbouring endogenous KoRV in their genome led to a significant increase in their circulating anti-KoRV IgG levels. This is a very important finding as it was previously argued that vaccinated koalas harbouring endogenous KoRV may behave similarly to pigs, which are tolerant to the porcine endogenous retroviruses^12^. Our 2019 study14 showed that the situation with koalas appears to be similar to cats and humans, which produce a robust antibody response against their endogenous retroviruses, FeLV and human endogenous retrovirus K (HERV-K), respectively. The antibody responses that we observed in our current study further confirm that endogenously infected koalas are not tolerant and can indeed generate specific anti-KoRV antibodies. The mechanism by which an animal produces an immune response to an endogenous retrovirus remains unclear. The recent discovery of piwi-interacting RNAs in koalas, which have the ability to silence the expression of endogenous retroviral sequences during ontogenesis, may offer a possible explanation^18^.

Recent studies suggest that KoRV-A sits at an intersection between a fully endogenized retrovirus and an exogenous retrovirus through its ability to produce infectious particles in the koala^19^. KoRV-A-directed immune response in natural infection has also been previously established, thus confirming the possibility for vaccine development^14^. Similar to observations from our current study, ongoing studies in humans have shown that HERV-K-directed immune responses can serve as a basis for vaccine development against HERV-K-associated cancers^20,21^. For instance, HERV-K Env protein was shown to offer both prophylactic and therapeutic benefits against tumour growth in mice.

The development of therapeutic vaccines is a major research focus for human viral infections, such as HIV and human papillomavirus (HPV)^22,23^. The aim of a therapeutic vaccine is to control an already established infection by enhancing the activity of the immune system against a target protein without inducing self-antibody or T cell-mediated inflammation^24^. For a viral infection, such as HIV, a very important indicator of therapeutic benefit for a vaccine is a significant and sustained reduction in circulating viral load^22^. In the current study, we observed a complete clearance of circulating KoRV-A in the three koalas that had detectable levels prior to vaccination. It should be noted that the vaccine antigen was designed based on a KoRV-A Env protein. We also observed a significant reduction in the expression of KoRV-B, suggesting that a KoRV-A Env protein-based vaccine can offer cross-protection against other KoRV subtypes. A possible explanation for this observation may be found in the high degree of similarity between both KoRV-A and -B subtypes. Whereas KoRV-A and KoRV-B are known to use different receptors to infect cells, both subtypes still share >95% of their Env protein sequences. This provides a substantial platform for epitope cross-recognition and subsequent cross-protection, especially if the antibodies are neutralising in nature. This finding is highly encouraging, as it suggests that a KoRV-A Env based vaccine may offer protection against other KoRV subtypes.

In contrast to our results, no beneficial effect on anti-FeLV antibodies or plasma viral load was reported in a FeLV vaccine trial where two licensed prophylactic FeLV vaccines were tested for possible therapeutic potentials in persistently infected cats^25^. Similar to our findings, however, significant progress is being made in the development of therapeutic vaccines against HPV and HIV, although none has been approved for clinical use yet^23,26^. Reduction in viral load is a desired outcome because disease progression, as well as rate of transmission, have been shown to have a positive association with viral load^27^. Observational studies in koalas reported that a significant increase in KoRV viral load is linked with KoRV-associated lymphoma and leukaemia^2^. Recent studies have also shown that the presence of the exogenous KoRV-B strain is linked with susceptibility to chlamydia disease^3,11^. The reduction in viral load observed in this study may therefore be important in the control of KoRV-associated diseases.

An important finding in our study was the observed increase in neutralising antibody titres post-vaccination. A similar observation was previously reported in koalas that are either KoRV negative or those believed to be exogenously infected with KoRV following vaccination with a recombinant KoRV Env protein^13^. Our study is also supported by previous studies which showed the ability of the Env protein of several retroviruses to induce neutralising antibodies upon vaccination^28,29^. Although not tested for, due to assay limitations, we suspect that the antibodies produced following vaccination are cross-neutralising in nature, as shown in the reduction in viral load of other KoRV subtypes post-vaccination. Whereas therapeutic vaccines are thought to act mainly through the induction of cellular immunity^30^, our data suggest that broadly neutralising antibodies may also have a critical role to play in the efficacy of therapeutic vaccines.

The Env proteins of several retroviruses have been shown to contain both immunogenic and neutralising epitopes, thus making the Env protein a suitable vaccine antigen against retroviral infections^31,32^. In our study, we show that the KoRV-A Env protein contains highly immunogenic regions capable of stimulating protective immune response as measured by induction of neutralising antibodies and reduction in viral load in koalas harbouring endogenous KoRV. In particular, our study identified three vaccine-induced epitopes, as well as four vaccine-enhanced epitopes, with antibody recognition lasting up to 6 months post-vaccination. This finding could prove to be important in the design of peptide vaccines that induce long-lasting antibodies in koalas. We also show that antibodies directed against the KoRV-A Env protein cross-react with both the variable and conserved domains on the RBD of KoRV-B Env protein. The first requirement for a broadly neutralising antibody is for it to be broadly reactive^33^. Though not tested for neutralising activity, the presence of cross-reactive antibodies may explain our observation of reduction in expressed KoRV-B viral load following vaccination with a recombinant KoRV-A Env protein. This finding is highly encouraging for the development of a vaccine that may offer protection against multiple KoRV subtypes.

The impact of polymorphisms in host genetics on humoral immune response to vaccination has been well documented in humans^34^. In fact, it has been proposed that vaccine efficacy can be predicted using host genetic information, thus leading to personalised vaccines^35^. Since such information does not exist yet in the koala, this study aimed to bridge the gap by relating immunogenetic variation in our vaccinated koalas to antibody response following vaccination. We found significant immunogenetic variation in the koalas in this trial, as no two koalas had identical alleles for all the assayed MHC class I or class II genes. Due to this genetic variation, and the fact that nine out of ten koalas responded adequately to vaccination, we were unable to determine if any particular set of MHC alleles was associated with vaccine response. Though this observation is limited by sample size, it is highly encouraging considering that koalas are outbred, and any vaccine developed for wild koalas must be able to elicit appropriate immune response regardless of the genetic background of the individual koala.

The long-term survival of the koala is under threat from infectious disease agents such as *Chlamydia* and KoRV. KoRV is present as an exogenous and or endogenous infection in 100% of koalas from Queensland and New South Wales. This study showed that koalas harbouring endogenous KoRV can benefit from prophylactic vaccination against KoRV using a recombinant KoRV-A Env protein and that the mechanism of this protection might possibly be through the boosting of natural anti-KoRV antibodies and expanding the breadth of the recognised epitopes. We also show that the vaccine potentially has the capacity to offer cross-protection against multiple KoRV subtypes. This work lays a solid foundation for continued KoRV vaccine development in koalas.

## Methods

### Recombinant Env protein preparation

Recombinant Env protein used for vaccination was prepared as previously described^13^. In brief, KoRV-A Env protein sequence (GenBank accession number: AHY24807.1) was synthesised and cloned into pGEX-4T1 and transformed into *Escherichia coli*. The transformed cells were cultured in rich Luria-Bertani Medium containing 30 mg/ml of filtered sterile Ampicillin, then induced by adding 0.3 mM isopropyl b-D-1-thiogalactopyranoside (Sigma, St Louis, MO, USA) with subsequent incubation for 3 h at 250 rpm at 37 °C. The culture suspension was centrifuged at 5000 × *g* for 20 min at 4 °C, followed by lysing of the cells. The supernatant from the lysed cells was filtered through 0.22-μM syringe filter and used for affinity purification chromatography. The purified protein was desalted and concentrated by ultrafiltration.

### Animals and immunization

Ten healthy male koalas aged between 2 and 6 years, bred and housed at the Lone Pine Koala Sanctuary, Brisbane, Queensland, Australia were used in this trial. All koalas were assessed by a veterinarian and certified healthy before recruitment into the trial. All koalas were vaccinated with 50 µg recombinant KoRV Env protein vaccine along with a Tri-adjuvant. The Tri-adjuvant consisted of 250 µg polyphosphazine, 500 µg host defense peptide 1002, and 250 µg poly I:C (Vaccine and Infectious Disease Organization, Saskatchewan, Canada). A booster shot was administered to all koalas four weeks after the initial vaccination. Each vaccine dose was prepared as per Olagoke et al.^13^. Blood samples (2–3 mL) were collected prior to vaccination and at 4, 8, 12 and 24 weeks post-vaccination from the cephalic vein into EDTA collection tubes (Interpath Services). Plasma samples were obtained from whole blood for use in serology or viral load quantification. Plasma samples (300 µL) for quantification of KoRV viral load were preserved in 900 µL RNALater. All samples were stored at −80 °C until use. Throughout the trial, all koalas were assessed individually on a daily basis for their health. All animal work reported in this study was approved by University of the Sunshine Coast Animal Ethics Committee (ANA18138). This study was conducted in compliance with approved institutional guidelines.

### KoRV genotyping and viral load quantification

Circulating KoRV viral load in plasma was quantified using previously described primers and cycling conditions^36^. Viral RNA was extracted from plasma stored in RNALater using QIAamp Viral RNA kit (Qiagen). RNA samples treated with TURBO-DNA-free (Thermo Fisher Scientific) were used for cDNA synthesis using iScript cDNA synthesis kit (Bio-Rad). All reactions were carried out as per the manufacturer’s instructions. Standards of known concentration (10^7^–10^1^) of each target KoRV subtype were prepared for each run and the results from the koala samples were normalized against the standard curve. qPCR reactions were performed using a CFX 96 Touch System (Bio-Rad, Australia) under the conditions described by Quigley et al.^36^.

### Anti-KoRV IgG ELISA

Anti-KoRV IgG levels expressed as endpoint titres were determined as previously described^13^. In brief, 96-well ELISA plates (Clear Flat-Bottom Immuno Nonsterile; Thermo Fisher Scientific) were coated with 1 µg recombinant KoRV Env protein and incubated overnight at 4 °C. Plasma samples serially diluted two-fold, starting at 1:200, were added to the 96-well plates and incubated at 37 °C for one hour. This was followed by the addition of sheep anti-koala IgG as secondary antibody and donkey anti-sheep as detecting antibody. The plates were washed after each step with PBS containing 0.05% Tween-20. Finally, TMB substrate was added to the plates for colour development. The reaction stopped using 1 M H_2_SO_4_ and colour development was measured at 450 nm. Endpoint titre was calculated as described in Olagoke et al.^14^.

### KoRV in vitro neutralisation assay

To determine the capacity of koala plasma samples to neutralise KoRV virus particles in an in vitro assay, we adapted a described protocol^13^. HEK 293T cells (ATCC, CRL-11268) were seeded in 96-well plates (Greiner bio one; Interpath, West Heidelberg, Australia) and grown until they reached around 50% confluence. The media was subsequently removed and replaced with a mix of koala plasma serially diluted in replication competent KoRV-A (supplied by Dr Maribeth Eiden, NIH). Prior to addition to the cells, the plasma-KoRV mix was pre-incubated for 30 min at 37 °C. The cell culture was subsequently incubated for 65 h at 37 °C under 5% CO_2_. A parallel culture with no koala plasma was also conducted. This allowed the viral titre to be determined in the presence or absence of koala plasma. Following incubation, genomic DNA was extracted from the cells using QiaAmp DNA mini kit (Qiagen). KoRV-A proviral load was subsequently quantified using the method described above. Neutralisation was defined as the reciprocal of the plasma dilution that resulted in 50% reduction in KoRV-A proviral integration when compared to the no plasma control.

### Epitope mapping of KoRV Env protein

Epitope mapping was performed using a total of 138 KoRV-A and 30 KoRV-B overlapping synthetic peptides (15 mer, 4 amino acids offset). The KoRV-A peptides were designed to span the near full-length recombinant KoRV-A Env protein, minus the first 100 amino acids. This sequence represents the sequence used to make the recombinant protein for vaccination. The KoRV-B sequence was designed to span only the RBD of KoRV-B as this is the region that contains most (~90%) of the sequence variation between KoRV-A and KoRV-B. The peptides were synthesised by JPT (Berlin, Germany) and anchored PepStar^TM^ microarrays. To prevent false negatives as a result of sterical hindrances, an optimised hydrophilic linker moiety was inserted between the peptide and the glass surface. Full-length human and sheep IgGs were co-immobilized on microarray slides as assay controls. Additional sequences were included in the peptide library by JPT as internal printing process controls. Koala plasma samples diluted 1:200 in a blocking buffer were incubated on the microarray slide for 1 h at 30 °C. Subsequently, sheep anti-koala IgG diluted 1:5000 in a blocking buffer was added and incubated for 1 h. A tertiary fluorescently labelled anti-sheep-IgG antibody at 0.1 μg/ml was added into the corresponding wells and left to react for another hour. The slides were subsequently scanned using Axon Genepix Scanner 4300 SL50 (Molecular Devices, USA) at 635 nm to obtain fluorescence intensity profiles. Resulting images were quantified to yield a mean pixel value for each peptide using spot-recognition software GenePix. To identify which amino acids were responsible for peptide responses, an in-house scoring system was used. In brief, the fluorescence intensity from a peptide was ascribed to each amino acid in the peptide. Where peptides overlapped, the fluorescent intensity for each peptide was averaged and assigned to the overlapping amino acids. Amino acids with fluorescent intensities above 2000 (background + 3X SD) was described as reactive.

### MHC allele assignment

Two MHC class I (UA and UC) and four MHC class II (DAb, DBb, DCb, DMb) genes were PCR amplified based on published primer sets^15,16^. Amplicons were deep sequenced at the Ramaciotti Centre for Genomics (Australia). Forward and reverse amplicons were filtered and trimmed with cutadapt^37^, merged with FLASH^38^, and reduced to unique sequences with prinseq^39^. Unique MHC sequences were BLAST searched against known MHC gene sequences to allocate allele numbers.

### Reporting summary

Further information on experimental design is available in the Nature Research Reporting Summary linked to this article.

## Supplementary information

Supplementary Information

Reporting Summary

## Data Availability

The data that support the findings of this study are available from the authors on request.
