# Supplementary material for: Therapeutic vaccination of koalas harbouring endogenous koala retrovirus (KoRV) improves antibody responses and reduces circulating viral load
Source: NPJ Vaccines. 2020 Jul 16;5:60. doi: 10.1038/s41541-020-0210-9 (PMC7367292; doi:10.1038/s41541-020-0210-9)

<u>Supplementary figures for:</u>

Therapeutic vaccination of koalas harbouring endogenous koala retrovirus (KoRV) improves antibody responses and reduces circulating viral load

O Olagoke[1], BL Quigley[1], F Hemmatzadeh[2], G Tzipori[3] and P Timms[1]

[1]Genecology Research Center, Faculty of Science, Health, Education and Engineering, University of the Sunshine Coast, 90 Sippy Downs Drive, Sippy Downs, QLD 4556, Australia; [2]School of Animal and Veterinary Sciences, The University of Adelaide, Roseworthy, SA 5371, Australia and [3]Lone Pine Koala Sanctuary, Fig Tree Pocket, Queensland, Australia. Correspondence: P Timms (ptimms@usc.edu.au)

Supplementary Figure 1: Sequence alignment of KoRV-A and KoRV-B Env proteins

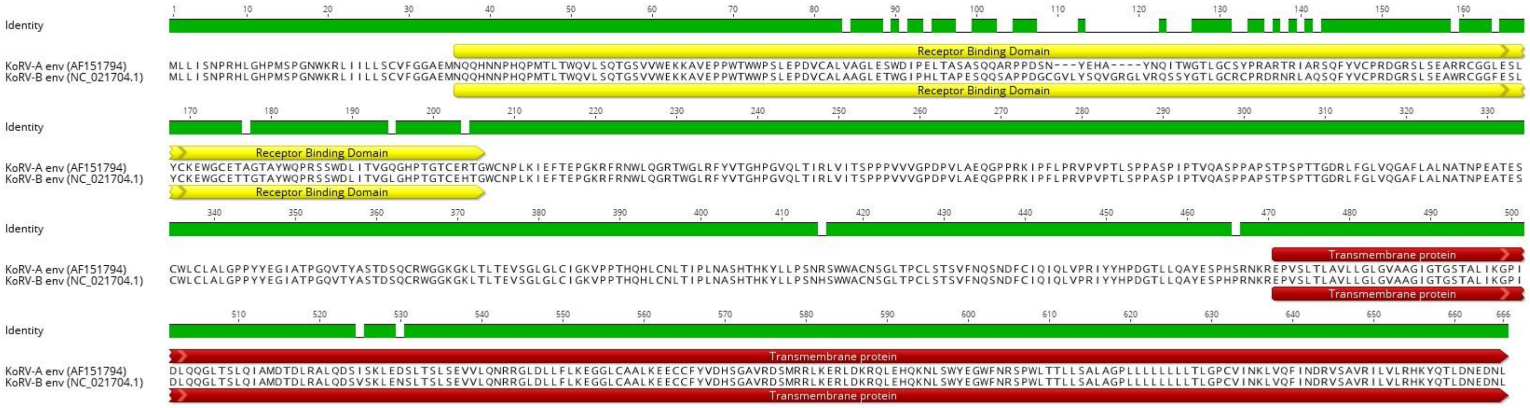

Supplementary Figure 2: Changes in the expression of KoRV-D and KoRV-F in koalas harbouring endogenous KoRV following vaccination. The coloured shapes represent individual koalas with detectable KoRV expression. These are subsequently represented with black circles when KoRV expression becomes undetectable.

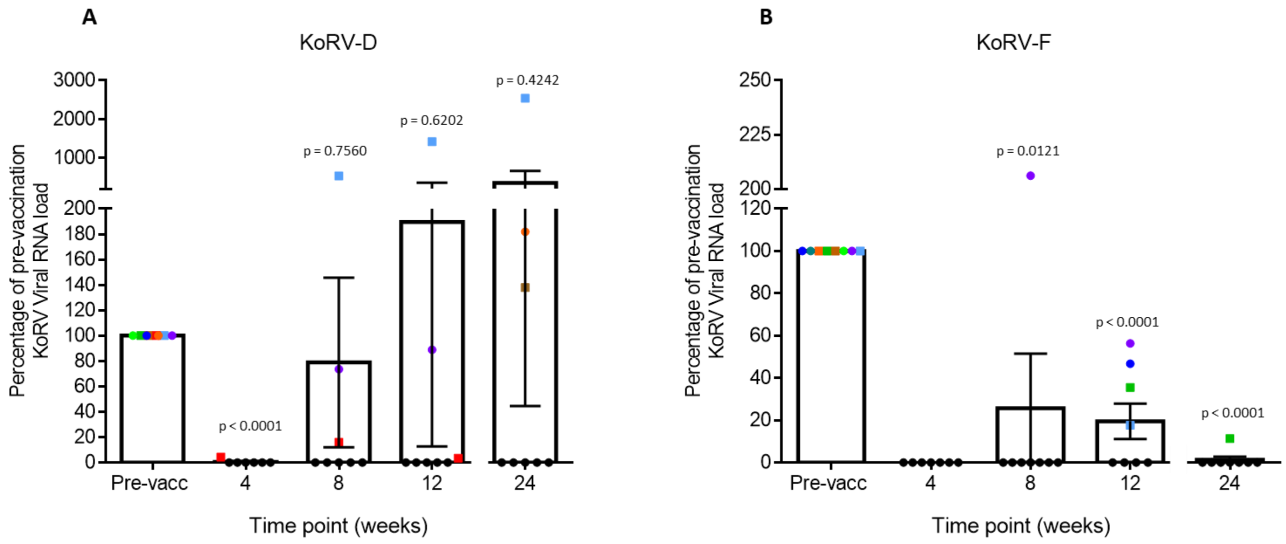

Supplement: Supplementary file 1 — Supplementary Information [file 41541_2020_210_MOESM1_ESM.pdf]
